# Menopause care for diverse communities: a qualitative study of GP clinician experiences

**DOI:** 10.3399/BJGP.2024.0780

**Published:** 2025-10-21

**Authors:** Claire Mann, Lisa Shah, Abi Eccles, Sabrina Keating, Jeremy Dale, Patricia Apenteng, Sarah Hillman

**Affiliations:** 1 Warwick Medical School, Coventry, UK; 2 Nuffield Department of Primary Care Health Sciences, University of Oxford, Oxford, UK; 3 University of Birmingham, Department of Applied Health Sciences, Birmingham, UK; 4 University of Birmingham, School of Health Sciences, Birmingham, UK

**Keywords:** diverse communities, ethnic minorities, general practice, health inequalities, hormone replacement therapy, menopause

## Abstract

**Background:**

In the UK, there is increasing public awareness of menopause. However, there remain inequalities in its treatment, with lower hormone replacement therapy (HRT) prescribing in areas of social deprivation. Little is known about how healthcare professionals (HCPs) view and understand this inequality.

**Aim:**

To explore barriers to, and facilitators of, the provision of menopause care in diverse communities.

**Design and setting:**

Qualitative, semi-structured interviews were conducted with HCPs across central England.

**Method:**

The authors purposively sampled 15 HCPs working in patient-facing roles in areas of high deprivation. An interview schedule was developed, and 11 individual interviews and one focus group were conducted between January and March 2024. The data were subject to team-based iterative thematic analysis.

**Results:**

Data were categorised into three key themes: the context of contemporary primary care, delivering menopause care, and limitations of the current approach to menopause care. HCPs reported that awareness of menopause and requests for HRT were increasing, but cultural and ethnic differences were perceived as affecting whether women sought menopause care and/or HRT from their GP. HCPs believed women had high expectations of HRT and felt that discussions around realistic expectations were important. They also emphasised: the difficulty of remaining up to date on menopause care; there being limited time, resources, and ability to refer to specialists; the impact of patients’ requests for testosterone; the need for targeted, culturally sensitive patient outreach initiatives and education; and the need for dedicated training for HCPs.

**Conclusion:**

HCPs believed that differences in levels of menopause care across diverse populations that experienced health inequalities reflected differing demands from communities, as well as a lack of time and funding to provide targeted community-based education on menopause and its treatment. Future work to improve menopause care should include additional research as well as culturally sensitive and targeted health education for patients and HCPs.

## How this fits in

Evidence shows that women from minoritised ethnic groups are less likely to access menopause care and support than White women. This study elicited healthcare professionals’ (HCPs’) perspectives on the barriers to, and facilitators of, effective menopause care for these women. Cultural and ethnic differences were perceived as barriers to seeking advice and/or treatment, and limited time and resources were seen as barriers to delivering effective care.

Targeted, culturally sensitive outreach initiatives and health education for patients, along with specific training for HCPs, could help to reduce health inequalities in menopause care, but further research into women’s experiences of receiving care is needed to inform this.

## Introduction

Menopause awareness is undergoing unprecedented change in the UK, not least due to increasing visibility in mainstream media.^1–3^ This awareness has been reflected in an increasing uptake of hormone replacement therapy (HRT) prescriptions nationally.^4,5^ However, inequalities persist in menopause care internationally: it was recently found that African American women had lower use of menopause treatments,^6^ despite evidence of them experiencing more severe symptoms^7,8^ and, in the UK, a difference of up to 30% between rates of HRT prescribing in different geographical regions associated with levels of deprivation was recently reported.^9^

There is an established link between geographical regions identified as deprived and the ethnic diversity of populations in those areas. Furthermore, it has been found that approximately five times fewer Black women (5.2%) and four times fewer Asian women (6.2%) were prescribed HRT compared with White women (23.3%), and nearly twice as many women from the most affluent groups (deciles 9 and 10 ) were prescribed HRT (23%) compared with those from the most deprived groups (deciles 1 and 2) (12%).^10^

A recent qualitative study investigating GPs’ perceptions on factors affecting menopause-related health-seeking behaviours in women from minoritised ethnic groups provided insights into the factors that affect uptake of menopause care. It identified that GPs felt they needed more time for in-depth communication and education with women from minoritised ethnic groups.^11^ That study focused on factors affecting the take-up of menopause care in minoritised ethnic groups; the study presented here was undertaken in deprived communities and, therefore, takes place where the intersectionality of health inequalities exists. This study is crucial to understanding primary care clinicians’ experiences of providing good menopause care in such communities, including care to a range of women from minoritised ethnic groups. It, therefore, builds on earlier work by exploring frontline clinicians’ perspectives on delivering good menopause care to women from diverse communities who are likely to experience health inequalities and have a lower uptake of HRT.^8^

A recent realist review^12^ suggested that general practice can contribute to reducing health inequalities. The authors outlined some of the difficulties of working in UK general practice in areas of deprivation, where there are fewer resources and higher demands, and suggested that existing evidence provides little guidance on how this reduction in health inequalities can practically be achieved. The study presented here seeks to explore how general practice can reduce health inequalities around menopause, and to answer the following primary research question: what are the barriers to, and facilitators of, menopause care in diverse communities from the perspectives of frontline clinicians in English primary care?

## Method

The authors purposively sampled 15 primary healthcare professionals (HCPs) working in areas of high deprivation, where health inequalities are known to be prevalent. Any HCP delivering menopause care to women in a primary care site in a deprived area (Index of Multiple Deprivation [IMD] deciles 1–6) was eligible to participate. The study was advertised through the National Institute for Health and Care Research (NIHR) Clinical Research Network West Midlands and local primary care network groups. Volunteer participants were invited to express their interest and provide their professional role, experience, and location for an eligibility check. Participants with a range of ages, experience, and roles were included. Eligible participants were provided with an information sheet, consent form, and choice of individual interview or focus group conducted online or via telephone.

The female research team comprised a range of professional backgrounds, including academic GPs, a GP trainee and academic clinical fellow, social science researchers, and patient and public involvement (PPI) representatives for three different ethnic groups. The interview schedule (Supplementary Data S1) was developed by the research team, with input from key stakeholders — including clinicians working in areas of deprivation and PPI representatives — during a workshop that took place in July 2023. Co-designed to explore HCPs’ experiences of providing menopause care and prescribing HRT in deprived communities, it was semi-structured around topic areas, with prompts to allow for free-flowing discussions. Questions explored experiences of providing menopause care in deprived communities and attitudes towards menopause care.

In total, 11 individual interviews and one focus group (*n* = 4 participants) were conducted by four members of the research team via Microsoft Teams during January, February, and March 2024. All interviews were audio-recorded and transcribed verbatim before data were transferred to NVivo (version 11) for analysis.

Interview data from two interviews were initially analysed by four researchers independently, using the one-sheet-of-paper method, a qualitative technique that facilitates thematic mapping and comparison across cases.^13^ A reflexive analysis workshop was undertaken with the same researchers and wider research team; through discussions, a coding framework was produced to respond to the research question based on the emergent data. The full dataset was then analysed by two researchers utilising the coding framework, and the findings were discussed and refined at further iterative analysis meetings with the whole research team.

## Results

### Participants

Participants were HCPs with different roles: GPs (*n* = 8), trainee GPs (*n* = 4), nurses (*n* = 2), and a physician’s associate (*n* = 1). Participants represented a range of ethnic groups — White (*n* = 8), Asian (*n* = 5), and Black or mixed Black (*n* = 2) — and included males (*n* = 2) and females (*n* = 13) with a wide range of experience (from trainee to ≥20 years’ experience). Table 1 details participants’ characteristics. Interviews lasted 30–90 minutes.

**Table 1.** Participant characteristics

Identifier	Job title	Experience, years	Gender	Area	Practice population, *n*	IMD decile
HCP01	GP	18	F	Coventry	>12 000	2
HCP02	Physician associate	22	F	Coventry	>12 000	2
HCP03	GP	≥25	F	Oxford	>12 000	6
HCP04	GP	5	M	Birmingham	6000–12 000	2
HCP05	GP	≥20	F	Bradford	>12 000	2
HCP06	GP	≥40	F	Coventry	<6000	6
HCP07	GP (plus clinical lead community gynaecology)	20	F	Birmingham	>12 000	2
HCP08	Nurse (specialist nurse practitioner)	≥20	F	Cambridgeshire	6000–12000	6
HCP09	Nurse (lead practice and primary care network nurse)	35	F	Warwick	>12 000	5
HCP10	GP trainee	ST3	F	Nottingham	6000–12 000	6
HCP11	GP trainee	ST2	M	Nottingham	>12 000	3
HCP12	GP trainee	ST3	F	Nottingham	>12 000	2
HCP13	GP trainee	ST2	F	Nottingham	6000–12 000	3
HCP14	GP	≥20	F	Mansfield	>12 000	5
HCP15	GP	5	F	Sheffield	6000–12 000	1

a Deciles rank 1–10 (1 = most deprivation; 10 = least deprivation). F = female. HCP = healthcare professional. IMD = Index of Multiple Deprivation. M = male. ST = specialty training.

### Themes

The authors categorised the barriers to, and facilitators of, menopause care into three key themes:

- the context of contemporary care in England;
- current menopause care; and
- limitations of the current model.

#### The context of contemporary care in England

This first section outlines the broad context in which HCPs provided menopause care and the experiences of women who sought support for symptoms; it also recognised that many women did not seek menopause care in general practice at all.

Participants discussed awareness of menopause and how that impacts patient presentation. They talked of their patients’ increasing awareness of menopause and HRT as a treatment option. All mentioned the impact of celebrities raising awareness of menopause and increasing information in the news and on social media. As one HCP commented:

*‘More recently, they've come particularly because they're so aware of menopause symptoms in the general population. I think you'll probably hear it across your interviews, but the Davina McCall programmes are strongly influencing people to come.’* (HCP01)

Participants understood that patients perceived access to GPs to be challenging and suggested that some women experienced symptoms for a long time before accessing support:

*‘Most of them have been suffering with symptoms for at least, sort of, three to four months before they come, and many substantially longer.’* (HCP01)

All participants stated having seen many women who had self-diagnosed; this was felt to be a change evident in the last 5–10 years, reflecting greater awareness and understanding about menopause:

*‘I had a patient the other week who came with, like, she had all those little notes in a booklet, that she'd read. You know, she’s very well informed, and patients will be very well informed about their symptoms, and they'll be tracking —* [using] *those tracking apps and things — with their periods and things like that.’* (HCP14)

In spite of this, participants recognised that there were still some women who presented with typical symptoms, but were unaware that they could be menopause related:

*‘The people that I tend to see, because of the socioeconomic class, the vast majority of people come in with a whole list of stuff. And I just think, "Oh, this sounds like you're perimenopausal or menopausal." And then they're like, "Well, I did think maybe, but then, actually ..." Or, or it comes as a complete surprise.’* (HCP11)

Some participants noted this as a cultural difference.

Several participants also reported some cultural differences in presentation:

*‘I would say most of the women we deal with are women who are, kind of, in their late forties, fifties, who are still generally working and maybe struggling with that. But it’s predominantly, sort of, late forties, fifties, up to sixty-plus. But they generally tend to be White British.’* (HCP08)

This indicated that the increased awareness driving appointments may not have been spread evenly across communities.

HCPs also recognised the risk of making assumptions about how women with menopausal symptoms might present:

*‘Am I beginning to expect that everyone who wants to talk about menopause is, themselves, going to introduce it and, themselves, going to talk about it? And, actually, if that’s not the case, then if we don't, kind of, maybe if I don't actively recognise that, might I be missing opportunities? And then maybe some inequalities could come up in that way.’* (HCP03)

Participants recognised that there were still many patients who lived with menopause-related symptoms and did not present to primary care:

*‘I think a lot of women probably don't just present because they just feel it’s something that they should be tolerating or just going through as a part of ageing.’* (HCP02)

#### Current menopause care

This second section focuses on the experiences of HCPs who provided menopause care in areas of deprivation, within the context outlined in the first section. They focused on delivering safe and satisfactory consultations to women, with recognition of different cultural needs and identification of the systemic barriers to providing care.

Participants agreed that taking a good history was vital to understanding whether a patient was experiencing menopausal symptoms and devising a management strategy. They discussed the importance of differential diagnoses when advising women with potential menopause symptoms. GPs discussed potential overlap with physical health issues:

*‘If they're complaining about night sweats, but they're heavy smokers,* [you might be] *thinking* [...] *“Could this be a cancer?” Don't just assume it’s menopause.’* (HCP08)

GPs also discussed potential overlap between menopause symptoms and mental health issues:

*‘There’s a big, sort of, crossover with mental health problems, and anxiety in that age, anxiety and depression in that age group. And whether to attribute that to menopause, or whether that is just anxiety and depression, and needs treating as such, I think is quite difficult. And so, I've certainly seen anxiety as a result* [...] *of menopause being misdiagnosed.’* (HCP10)

Due to difficulties with diagnosis, some participants had seen women who had not had a discussion about menopause, despite previously having presented to other clinicians on multiple occasions with symptoms that were potentially menopause related. All participants agreed that:

- menopause care should focus on understanding patients’ needs and expectations;
- women’s experiences and symptoms could vary greatly; and
- those experiencing health inequalities might not anticipate menopause or might experience other overlapping morbidities and communication issues that added barriers to presentation.

All participants agreed that a good consultation about HRT includes a discussion about its risks and benefits. Several participants suggested that many patients had very high, and unrealistic, expectations of how HRT could help them:

*‘I saw more and more of these people, who, ultimately, were disappointed with HRT because it didn't deliver what they were hoping it was going to deliver. And, therefore, I got a little bit more savvy doing the consultations to try and see what it was that was bringing them for the consultation* [...] *But they weren't coming with acute presentations. A lot of it was social situations and emotional situations. And, you know, HRT was not* [beneficial]*, and, and in a way, that was very disappointing for me and very disappointing for them, because* [...] *it was, like, their last-chance saloon for happiness, and then it didn't materialise.’* (HCP08)

They noted that patients had to be counselled to have realistic expectations. Several interviewees reported that, in most cases where expectations were managed, HRT had had a positive impact:

*‘I think for the ladies that are on HRT, as long as they’re told it’s not an immediate fix, that sometimes we’ll have to tweak things, it might take a little bit of time to get them where we want them to be,* [as long as we] *temper their expectations immediately, I think, I think it’s* [HRT]*, it’s really great, and I think the vast majority are delighted with it, to be fair.’* (HCP09)

Participants reported that patient safety factors determined the treatment they offered, including the form and dose of the HRT prescribed, but some felt that there were a lot of HRT options and updates of which they had to keep abreast. Many participants reported having received requests for testosterone and some said they had been asked to prescribe off-licence doses of hormones:

*‘The other thing that we've had highlighted at one of our meetings is requests from private doctors to prescribe quite high-strength preparations of oestrogen, which we've had a, had a discussion about, and, and made a policy that* we're not *comfortable to prescribe off licence. And we'll make that clear to the patient, and the private doctor as well.’* (HCP04)

Dealing with these requests was felt to present an increasing stress on clinician capacity. Variance was reported in relation to approaches to testosterone based on geographical location and clinical experience:

*‘I mean, at the moment we haven't got a licensed preparation for women for testosterone so I think that has to be a barrier, because you have to tell people that it’s not a licensed thing when you prescribe the meds and you have to explain all that.’* (HCP07)

*‘I don’t start patients on testosterone a great deal of the time. I can count on my hands the amount of times I’ve done it.’* (HCP11)

*‘We've recently been able to start prescribing testosterone for low libido, with some guidance about how to do the bloods, and how to follow these patients up. I guess we have more clarification about when to do blood tests, and things. So yes, in that respect, that has really grown.’* (HCP14)

Participants agreed on the importance of continuity, and some attempted to schedule ongoing monitoring after HRT had been prescribed. In a context of overstretched resources, however, most suggested that they found continuity and personal follow-ups unachievable. As one HCP stated:

*‘The only thing is, sometimes we don't get to follow up with the same patient.’* (HCP02)

Participants accepted that some women would not choose to take HRT and wanted to discuss alternatives, such as over-the-counter medicines and lifestyle changes:

*‘For whatever reason — might not just be a contraindication, might be that they're happy to go with, you know, conservative lifestyle measures, herbal remedies — and for whatever reason* [... patients] *don't want to use HRT or something prescribed. Just, again, anecdotally, I just remember one lady saying, “Well, yeah, it’s the menopause, it’s gonna happen. And happened to my mum, and she got through it. So I’m sure I'll be fine.”’* (HCP04)

#### Limitations of the current model of menopause care

In this final section, the limitations of providing care, and why the current care model does not work for many people, are considered. Participants explored the needs of those experiencing health inequalities, and the barriers and facilitators to providing care to these groups.

Resource implications of meeting the needs of diverse and deprived communities were discussed; this included the provision of interpretation services:

*‘About thirty to fifty per cent of our consultations are in a foreign language so, we've got three interpreters in the practice at all times.’* (HCP15)

Some participants suggested that women presented and discussed menopause differently, depending on their cultural background. Difficulties in having meaningful conversations through an interpreter, which was sometimes a family member, were also highlighted:

*‘I have definitely spoken to patients who've talked about not having words for “menopause” or how it wasn't something that gets talked about. So there may be groups where there is stigma or a taboo.’* (HCP03)

When asked about the ethnic groups of women presenting for help, multiple participants reported that, in their experience, non-White women were less likely to present with symptoms:

*‘I can't remember the last time I spoke to anybody Black or Asian about HRT, which is interesting.’* (HCP10)

Participants also reported that they saw an overlap between affluence and awareness/understanding related to menopause, with more affluent women being more likely to self-diagnose and advocate:

*‘From the more deprived area, it tends to be something that you suggest to them, I think. You, sort of, say, “Could this be menopause?*” *And they say, “Oh, yeah, well, yeah, I am fifty. I suppose that could be what’s happening.*” *It’s like, so, we have a chat about it, and then that leads on to a chat about HRT. So, certainly, certainly* [there is] *a difference in, in the approach to seeking HRT between the two demographics, I’d say.’* (HCP11)

Participants reported that there were many patients with low levels of menopause awareness/understanding and that it was easier for the GP to support patients with higher levels of menopause awareness. When asked about this barrier, interviewees suggested that written information might not reach everyone and misinformation still exists; however, several also proposed that outreach and advocacy work in communities could help, and gave practical examples from their own work and networks. Examples of best practice are shown in Supplementary Box S1.

When asked about barriers to care, many participants recognised that access to GPs can be problematic:

*‘Can you get past the front desk? Do you have the, kind of, social capital, the confidence to get past the front desk to get into an appointment? Does the type of consultation make a difference? Is this something where, if it’s all remote, you might not feel* [heard]*?” I don't know the answer to that. But I guess those are things that I wonder about.’* (HCP03)

All participants suggested that time and capacity were the largest barriers to providing good menopause care:

*‘Time. So, the people I work with are brilliant. Primary care is so stretched at the moment. Stretched to capacity, stretched financially. We’re seeing our, our income reduce year on year, over the last few years. Particularly since COVID. I have not asked for more than ten-minute consultations* [for] *menopause, but I know that they* [GP partners] *would say “no”. Because it’s core GP work, it’s standard GP work. But you try and do a menopause consultation, even a follow-up, in ten minutes. It’s absolutely impossible.’* (HCP05)

More time and, often, multiple appointments were considered necessary to be able to provide good care to patients from minoritised ethnic groups; this was seen as a barrier in an environment with limited capacity:

*‘We haven't got the resources or the time to back it up. ’Cause, effectively, what we're doing by doing that is saying, “Oh, let’s just have a load of double-length interpreter consultations with our most experienced senior female staff”, who are already in demand. So I think we would, anything that we did, sort of, along these lines, would have to be really well financed.’* (HCP01)

*‘I mean part of the beauty of being a GP is that you develop that rapport with the patient and you can figure out what it is that they will most benefit from. But you need to have the luxury of getting to know the patient and the time to do that* [...] *so I think it comes back to the time.’* (HCP07)

Some participants reported receiving good support from secondary care for complicated cases. In contrast, others found it difficult to access the support needed from secondary care and suggested that the time required to access secondary care is a barrier to good menopause care:

*‘They* [Some patients] *should be under the care of a specialist in, in menopause. But then it’s so hard to access that, you know, you don't know when they're gonna be seen. It could be a year down the line, and patients want, want things done now, you know.’* (HCP04)

Participants made a range of suggestions based on their perceptions of the current context of menopause care and why the current care model doesn’t work for everyone. They suggested that general practice should have female GPs and nurses with a specialist interest in menopause care, and offer continuity of care where possible. Participants identified an increased need for menopause and HRT training, and awareness for all practice staff in order to facilitate the best care for all. They reported a need for up-to-date research and evidence, and targeted knowledge about specific communities:

*‘I’m a fan of continuity, so I like to see the ladies again … I like them to be able to come to me and say, you know, sorry doctor but the* [medicine] *did nothing and you need to do something more.’ (HCP01 )*

*‘The conversation about wider education that is* specific *to communities … then, there probab*ly *is something about training and expertise for people working in these areas, but very specifically targeted at, “Okay, how do you have a conversation with someone who’s really reluctant to have HRT? Or doesn't even understand it?*” *So, it’s not just generic training for menopause, it’s actually a bit more targeted, to say, “When you're working in these groups, and these areas, these are things that can be helpful. And these are the resources you can direct people to.*”’ (HCP15)

Participants suggested that, in contrast to the current climate of awareness, there is a clear gap in public awareness and education about the menopause in deprived communities, and suggested that women with greater understanding and awareness of menopause and HRT were more likely to receive good care. They described some of the resources to which they had signposted women, but also identified a gap in good-quality resources in different languages or those aimed at different communities and in easily digestible formats, such as video and audio, which would be effective alternatives to written resources. It was recognised that more-accessible information was needed in a range of languages and styles that are culturally relevant, authentic, and co-produced. Participants expressed that specific guidance on how to work with sub-groups of women might facilitate improvements in menopause care.

Many participants suggested that community outreach was important to reach specific groups, and several had evidence of existing positive work in this arena, including the benefits of learning in community groups. Some examples included working with specific minoritised community groups, and using mechanisms — such as family learning — to share education with, and through, multigenerational families. These were individual cases of excellence, and not linked to any projects working at scale; examples are outlined in Supplementary Table S1.

## Discussion

### Summary

HCPs reported a surge in demand for menopause support but, due to overlap with symptoms of other physical and mental health issues, cautioned against the risk of assuming symptoms were always related to menopause. They viewed the recent greater demand for menopause support and perceptions about HRT as reflecting increased awareness and education; however, they also recognised that there were still many women who had low awareness and did not seek healthcare support. In addition, it was noted that some women did not want HRT and also suggested that Black and Asian women were less likely to ask for it.

HCPs recognised that many women might want a more holistic approach, but current consultation approaches often did not deliver this. The findings also showed that some HCPs believed women had high expectations of HRT; as such, honest discussions around realistic expectations were felt to be important. New demands for testosterone and off-licence prescribing added increased pressure to the workload in primary care, as consultation time was needed to explain the need for prescribing within licence/guidance. Lack of available support from secondary care was also found to contribute to primary care workload and reduced the potential for good care (particularly for patients whose cases were complex). The biggest barriers to providing good care were time and resources, especially in terms of meeting the needs of specific communities, which were more resource intensive.

The research revealed some direct solutions to overcome the barriers of providing menopause care and HRT prescribing to all women. These included outreach work and education for women, as well as HCP training on: how different and diverse communities make sense of bodily changes, such as menopause; the cultural acceptability of seeking care and accepting treatment; and how women who are already struggling to survive prioritise their health differently.

### Strengths and limitations

This study has several strengths. First, the purposive sampling of HCPs from areas of deprivation means the findings are highly relevant to the target population, thereby enhancing the ecological validity of the study. The inclusion of a diverse range of HCP roles — including GPs, trainee GPs, nurses, and a physician’s associate — ensures representation of perspectives from a range of roles in differing primary healthcare settings that provide menopause care. The use of semi-structured interviews, alongside a focus group, allowed for an in-depth exploration of HCP experiences and the nuances of their interactions with women from minoritised ethnic groups and deprived areas. Furthermore, the systematic approach to data analysis, including the use of NVivo software and the collaborative development of the coding framework, ensured rigour and reliability in the thematic analysis.

The study also had some limitations. Although the sample was purposively selected, it was small and only included participants from central England; as such, it may not fully capture the diversity of experiences across different regions or in different healthcare settings. There may also have been an element of selection bias, which affected who participated.

### Comparison with existing literature

The findings reported here are consistent with recent literature.^11^ Participants reported increasing numbers of more affluent women proactively requesting menopause support and HRT prescriptions; this aligns with the evidence presented by others.^1,2,4,5,14^ However, the study also showed that participants experienced women with little awareness of menopause seeking support for symptoms, and it was recognised that many women did not seek menopause care in general practice at all. The authors found little published evidence around this topic as awareness has grown — there is a clear gap for research that is focused on women who are not accessing menopause support or seeking HRT prescriptions.

Participants suggested that there were some demographic groups, such as Black and Asian women, who were less likely to ask for menopause care or HRT prescriptions; this fits with evidence from the UK^11^ and the US.^6^

The findings demonstrate the importance of honest discussions around realistic expectations and the benefits of HRT. However, there is little recent published evidence evaluating patient expectations of HRT compared with outcomes, suggesting a need for more nuanced understanding of patient expectations and satisfaction.

The recent consideration of GP views on menopause care for women from minoritised ethnic groups^11^ demonstrated that time to communicate was a large barrier. The evidence presented here showed that new demands for testosterone and off-licence prescribing added further pressure to the primary care workload in relation to menopause. Furthermore, it also showed that limited access to secondary care support, due to capacity issues, reduced the potential for good care; this aligns with evidence of increasing demand^14,15^ and that limited available support from secondary care adds to demand in primary care.^16^

The study presented here emphasises the importance of the barrier of time, which was also identified in a study by Reeves *et al*.^8^ Although they focused on time in the consultation, the study presented here also showed the importance of longitudinal time, and the lack of it hindering the ability to offer continuity of care. The findings presented here concur with those from previously conducted work in this area,^11^ which showed that HCPs’ largest barriers to providing good care were time and resources, especially in terms of meeting the needs of specific communities, which commands more resources; this fits with the extensive evidence that shows how a lack of resources is linked to health inequalities ^17^

The authors’ research collated some direct solutions to overcome the barriers to providing excellent menopause care and HRT prescribing for all women. These included HCP training, along with outreach and education for women, which has been called for in a wide range of other studies.^11,18,19^

### Implications for research and practice

Recent research on patients’ expectations of HRT and how these compare with outcomes, as well as that on the importance of having honest discussions around realistic expectations and the benefits of HRT, is scant; further research on these areas could be undertaken. Furthermore, there is a potential risk of health inequalities being exacerbated in primary care: if HCPs model care around women who are increasingly proactive, they may miss potential menopause symptoms in those who are less proactive or have lower awareness. There is a clear need for further research with women from diverse communities in order to clearly understand their varying expectations and needs, the factors that may influence them, as well as to ascertain how they can be addressed in terms of service design and delivery.

Initiatives are being undertaken to improve menopause care in diverse communities, but these are not being done at scale. For large-scale change that addresses health inequalities, there must be an investment in culturally sensitive and targeted health education and resources, Dedicated training for HCPs must be included, as should investing in the time and staff required to provide continuity of education, care, and support for women throughout their menopause transition.
